# Impact of Xsens Technology on Analysis of Gait Deviation in Pre- and Post-surgical Total Knee Arthroplasty Patient

**DOI:** 10.7759/cureus.67539

**Published:** 2024-08-22

**Authors:** Neha M Chitlange, Deepali S Patil, H V Sharath, Raghumahanti Raghuveer, Moh'd Irshad Qureshi, Pratik Phansopkar

**Affiliations:** 1 Department of Musculoskeletal Physiotherapy, Ravi Nair Physiotherapy College, Center for Physiotherapy Education and Research (CAPER) Datta Meghe Institute of Higher Education and Research (DU), Wardha, IND; 2 Department of Paediatric Physiotherapy, Ravi Nair Physiotherapy College, Center for Physiotherapy Education and Research (CAPER) Datta Meghe Institute of Higher Education and Research (DU), Wardha, IND; 3 Department of Neuro-Physiotherapy, Ravi Nair Physiotherapy College, Center for Physiotherapy Education and Research (CAPER) Datta Meghe Institute of Higher Education and Research (DU), Wardha, IND

**Keywords:** angular velocity, center of mass, gait variables, xsens, total knee arthroplasty, knee osteoarthritis

## Abstract

Introduction

Osteoarthritis (OA) is a degenerative joint ailment that predominantly affects the knee and is most common in older adults. It destroys the surrounding tissues and cartilage. Following total knee arthroplasty (TKA), patients with end-stage knee OA can have long-term pain reduction and patient satisfaction, although certain functional limitations still exist. While TKA significantly reduces pain and improve function, many patients still experience abnormalities post-surgery, such as slower walking and reduced step-length. Gait analysis using technologies like the Xsens Motion Visualization and Navigation (MVN) system (Movella, Henderson, NV, USA)provides insights into these functional limitations, helping to assess knee mobility and predict changes in joint movements during activities.

Methods

Thirty-four people participated in a study done at the Centre for Advance Physiotherapy Education and Research, Ravi Nair Physiotherapy College, Wardha, both before and after total knee arthroplasty. Participants who ranged in age from 50 to 70 and had undergone unilateral TKA were included. Informed consent was obtained, and demographic data were collected. Participants walked a predetermined distance at their usual speed, and measurements of hip and knee angular velocity and center of mass were recorded for gait analysis.

Result

The study involved participants aged 50-70, with a mean age of 59.79 years and height ranging from 153.00 to 185.00 cm. Significant gait changes were noted pre- and post-total knee arthroplasty, including a decrease in walking speed from 0.72 to 0.55m/s and cadence from 105.06 to 82.86. Other parameters, such as step length and center of mass, also exhibited considerable differences, highlighting the impact of TKA on gait dynamics.

Conclusion

The study underscores the significant impact of total knee arthroplasty on gait mechanics and the value of advance of technologies like Xsens for assessing functional outcomes. While TKA provides pain relief and improved mobility, residual gait abnormalities persist, highlighting the need for tailored rehabilitation. Xsens technology enhances patient preparation, recovery tracking, and rehabilitation strategies, setting a new standard for gait analysis in orthopedic practice.

## Introduction

Osteoarthritis (OA) is the most prevalent degenerative joint disease affecting older persons worldwide, causing damage to surrounding tissues and cartilage [1]. Osteoarthritis of the knee is the most frequent joint disease in adults over 60. Within this group, symptomatic knee OA affects 10% of men and 13% of women [2]. In India, the estimated prevalence of OA knee was 28.7%, compared to a global average of 22.9% [3].

When end-stage knee osteoarthritis is accompanied by persistent discomfort and deformity in the knee, total knee arthroplasty (TKA) is a typical surgical surgery. Over 2.5 lakh TKA procedures are carried out in India each year, indicating a sharp rise in the procedure's prevalence [4]. By 2030, 3.5 million cases of primary TKA are expected to occur annually [5]. Despite long-term pain reduction and patient satisfaction, many patients who have total knee arthroplasty nevertheless report impairments and functional limits when compared to age-matched controls [6]. After total knee arthroplasty, patients walk 15% less slowly after a year than age-matched controls with no known knee pathology [7]. Individuals who had total knee replacement exhibited notably greater functional limitations throughout the timed stair-climbing task, as evidenced by a 50% reduced performance when compared to age-matched healthy persons [7].

Osteoarthritis in the knees frequently causes gait abnormalities in patients, such as slower walking, shorter steps, and prolonged single support times. Following surgery, total knee arthroplasty results in notable functional gains and a decrease in irregularities in gait, perhaps as a result of pain relief [8]. Step length imbalance, however, may still exist 15 days following surgery, potentially due to early post-operative crutch gait patterns [9]. Even after symptoms subside, limited knee extension following total knee arthroplasty is linked to a low Oxford Knee Score [10]. Nevertheless, limb symmetry in joint excursions and movement patterns improve until quadriceps strength equalizes a year following TKA [11]. Although TKA usually improves gait impairments, certain abnormalities may persist [12]. Facilitating the restoration of normal mobility and stability is the center of mass transfer from potentially changed pre-surgery to stabilized post-surgery, with the help of prosthesis alignment, improved gait, and muscular strength [13,14].

For gait analysis, Xsens technology- more specifically, the Xsens Motion Visualization and Navigation (MVN) system (Movella, Henderson, NV, USA)- is frequently used [15]. An inertial sensor system is used to precisely measure compartmental force values in instrumented total knee replacements [16]. Accurate measurement and analysis of human movement during different activities, such as steps, cadence, distance, joint angles, the centre of mass, acceleration, and gait cycle, are made possible by these wearable systems [15]. With the ability to predict alterations in knee joint moments during walking tests in senior individuals suffering from knee osteoarthritis, Xsens MVN offers comprehensive insights into gait patterns and facilitates the evaluation of knee mobility in daily activities [16].

## Materials and methods

Method

An observational study was conducted in the Ravi Nair Physiotherapy College in Wardha, Maharashtra, India, at the Centre for Advance Physiotherapy Education and Research (CAPER). The purpose of the study was explained to the participants, and their consent was obtained. The study included 34 people, both before and after total knee arthroplasty. An assessment procedure was carried out based on the inclusion and exclusion criteria (Table 1). The patient provided informed consent, and demographic information was gathered, including name, gender, age, height, weight, body mass index (BMI), and foot length. Participants in this study walk a predetermined distance (e.g., 10 meters) at their typical walking speed. The angular velocity (hip and knee) (x, y, and z-axis), the spatial and temporal characteristics, and the center of mass (x, y, and z-axis) of gait were recorded.

**Table 1:** Inclusion and exclusion criteria

Inclusion criteria	Exclusion criteria
50–70 years	older than 70 years old
male gender	individuals with cognitive difficulties
unilateral total knee replacement	bilateral total knee arthroplasty

Sample size

The sample size is determined using the following formula:

n = [Z α /22.P.(1-P)]/ d2, where Z α /2 is the level of significance at 5%, i.e., 95%; Confidence interval = 1.96αα; P = Prevalence of Total Knee Replacement Indian population = 4.23% = 0.042; d = Denied error of margin = 7% = 0.07; Total sample size = 34.

Statistical analysis

Statistical analysis was done by using descriptive and inferential statistics using Student’s paired t-test, and the software used in the study was SPSS 27.0 version (IBM Corp., Armonk, NY, USA). Pearson Chi-Square test was used to compare all qualitative variables at a significant level of p-value <0.05 for all variables.

Ethical considerations

This study was approved by the Institutional Ethics Committee, Datta Meghe Institute of Higher Education and Research (Ref no: DMIHER(DU)/IEC/2023/77) dated 20 December 2023. Participant confidentiality was maintained, and all procedures adhered to ethical guidelines for research.

## Results

**Figure 1:**
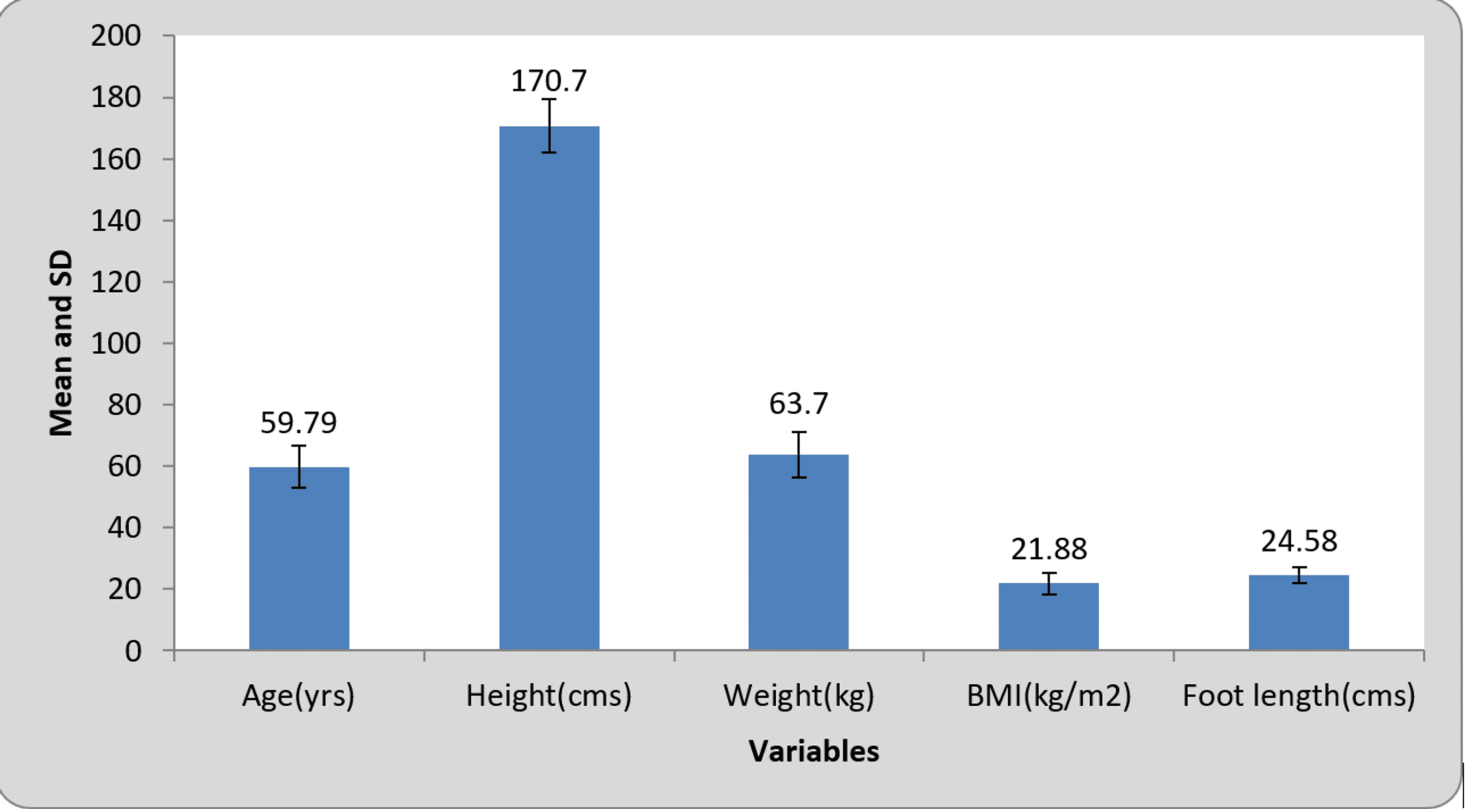
Comparison of gait variables at pre- and post-treatment SD: Standard deviation; p<0.05

In our study, according to inclusion criteria, the age group included was 50-70 years old. The minimum and maximum ages were 50.00 and 70.00 years, respectively. The mean age was 59.79, and the standard deviation was 6.85. The minimum and maximum heights of the patient were 153.00 and 185.00 cm. The mean height was 170.70, and the standard deviation was 8.74. The minimum and maximum weight of the patient were 50.00 and 77.00 kg, respectively. The mean weight was 170.70, and the standard deviation was 8.74. The minimum and maximum BMI of the patient were 16.40 and 31.20 kg/m2. The mean BMI was 21.88, and the standard deviation was 3.54. The minimum and maximum foot lengths of the patients were 20.00 and 29.00 cm. The mean foot length was 24.58, and the standard deviation was 2.52, as shown in Figure 1.

In a comparison of gait variables using the student's paired t-test, significant differences were observed across various parameters pre- and post-TKA. Notably, the patient's speed decreased significantly from pre-TKA (0.72±0.05) to post-TKA (0.55±0.03), with a mean difference of 0.17±0.05 (p<0.05). Cadence also decreased significantly post-TKA, with values of 105.06±4.93 pre-TKA and 82.86±5.28 post-TKA (mean difference: 22.20±7.11, p<0.05). Step length, step width, swing phase, stance phase, and midstance phase all showed statistically significant differences between pre- and post-TKA measurements (p<0.05), with varying mean differences. Moreover, the center of mass (COM) in all three axes (x, y, and z) exhibited significant differences pre- and post-TKA, indicating alterations in balance and stability. Additionally, the angular velocity of both the hip and knee displayed significant differences across all axes, underscoring changes in joint movement patterns following TKA. These findings collectively highlight the substantial impact of TKA on gait dynamics and joint kinematics. A comparison of gait variables, center of mass, and angular velocity of hip and knee pre- and post-total knee arthroplasty is shown in Table 1.

**Table 2:** Comparison of gait variables, center of mass, and angular velocity of hip and knee before and after TKA. TKA: total knee arthroplasty; COM: center of mass; AV: angular velocity; p-value <0.05

Components		Pre TKA		Post TKA		Mean Difference		t-value	p-value
Gait Variables	Speed (m/2)	0.72	±0.05	0.55	±0.03	0.17	±0.05	17.07	0.0001,S
	Cadence (steps/min)	105.06	±4.93	82.86	±5.28	22.20	±7.11	18.19	0.0001,S
	Step Length	7.69	±0.42	0.95	±0.34	6.73	±0.57	68.05	0.0001,S
	Step Width	0.39	±0.06	0.05	±0.02	0.33	±0.07	26.99	0.0001,S
	Swing Phase	3.85	±0.21	-4.09	±1.48	7.95	±1.48	31.28	0.0001,S
	Stance Phase	-2.42	±0.06	3.89	±0.30	6.32	±0.31	117.30	0.0001,S
	Mid Stance	-5.31	±0.14	1.43	±0.03	6.75	±0.15	254.82	0.0001,S
Centre of mass	COM X-Axis	0.71	±0.04	0.53	±0.01	0.17	±0.04	22.30	0.0001,S
	COM Y-Axis	0.30	±0.02	-0.07	±0.01	0.38	±0.02	75.71	0.0001,S
	COM Z-Axis	-0.02	±0.01	0.02	±0.02	-0.04	±0.03	9.70	0.0001,S
Angular Velocity Hip	Hip AV x	-0.47	±0.06	-0.15	±0.02	0.31	±0.05	35.44	0.0001,S
	Hip AV y	-1.85	±0.22	0.29	±0.02	2.15	±0.22	55.06	0.0001,S
	Hip Av z	0.29	±0.05	0.72	±0.27	0.42	±0.28	8.86	0.0001,S
Angular Velocity Knee	Knee AV x	-0.77	±0.27	-0.32	±0.03	0.44	±0.28	9.22	0.0001,S
	Knee AV y	2.84	±0.05	0.70	±0.02	2.13	±0.05	217.64	0.0001,S
	Knee Av z	-1.02	±0.03	1.27	±0.22	2.29	±0.23	57.80	0.0001,S

Statistical analysis was done by using descriptive and inferential statistics using Student’s paired t-test, and the software used in the study was SPSS 27.0 version, and p<0.05 is considered as the level of significance.

## Discussion

Using Xsens technology, the study offers insightful information about how TKA affects gait dynamics. The outcomes show a number of gait metrics that significantly improved after TKA. Significant improvements were demonstrated in speed, cadence, step length, step breadth, swing phase, stance phase, and mid-stance phase, underscoring the beneficial effects of surgery on mobility and functional performance. These results are consistent with earlier studies showing that TKA results in improved self-reported functioning and decreased irregularities in gait. It's interesting to note, nevertheless, that several gait abnormalities, like asymmetric step length and restricted knee extension during locomotion, persisted or even worsened after surgery.

Many changes have been noted in the mechanics of gait after total knee arthroplasty. They could be caused by things like muscular weakness, proprioceptive deficiencies, persistent pain, and prosthetic joint adaptation. Walking at a slower pace, having less knee flexion excursion, and having different knee moments in the sagittal plane are all signs that patients still struggle to achieve the best gait patterns even after surgery. Such deviations have an effect on the knee that is affected, but they may also put more strain on the joint on the other side, which raises questions regarding the course of the disease and the long-term health of the joints [17].

Significant COM shifts along the x, y, and z axes after TKA indicate greater stability and balance, which are probably related to less pain and better knee joint alignment. Comparably, after total knee arthroplasty, notable angular velocity alterations at the hip and knee joints show enhanced muscle function and joint mobility, possibly as a result of decreased joint stiffness and increased range of motion.

A thorough examination of gait dynamics, including angular velocity, center of mass dynamics, and spatial and temporal aspects, was made possible by the use of Xsens technology. With the use of this cutting-edge sensing technology, physicians can now schedule surgeries, monitor patients after surgery, and deliver individualized therapy with more accuracy and a thorough understanding of their patients' movement patterns. Beyond the scope of typical clinical examinations, real-world movement data collected during everyday activities offers a full picture of functional performance [18].

The limitations of the study were the following: Small sample size (34 patients) where results are still limited. The short follow-up period of two weeks after TKR in the study probably did not take into account all functional changes and adaptations that occur during rehabilitation. Further longitudinal research with larger samples and longer follow-up periods on gait patterns in rehabilitation is needed. Further results on gait after TKR are also needed, especially in proprioception, psychological problems, muscle strength and other patient-reported outcomes. Finally, research needs to investigate surgical techniques and rehabilitation processes, factors that can demonstrate the best course of action for functional recovery. Despite everything, the results seen here described in the study and obtained by Xsens technology are promising.

## Conclusions

TKR is the main suggestion for people suffering from end-stage knee osteoarthritis. However, the significant impact of arthroplasty on gait mechanics is poorly studied. The study was conducted using important inclusion criteria such as age range, minimum, mean and maximum ages (standard deviation), minimum, mean and maximum heights (standard deviation), minimum, mean and maximum weight (standard deviation), minimum, mean and maximum BMI (standard deviation) and finally the minimum, mean and maximum (standard deviation) foot lengths in comparison of gait variables before and after treatment. After comparing the gait variables using paired Student's t-test, significant differences were observed in the various parameters before and after TKR. Especially, the speed, cadence of the patient, which decreased significantly. There were also significant changes in step length, step width and swing phase, stance phase and mid-stance phase in gait. In turn, the center of mass (CM) in all three axes (x, y and z) showed significant differences pre- and post-TKR, indicating changes in balance and stability. Likewise, the angular velocity of the hip and knee showed statistically significant changes in all axes, also highlighting the changes in joint movement patterns after TKR. All these aspects are relevant and point to the impact of TKR on gait dynamics and joint kinematics. The results demonstrate that Xsens technology is capable of measuring all these parameters accurately and thus improving patient preparation, providing recovery tracking, mapping rehabilitation strategies and finally defining a new pattern of thinking for gait analysis in orthopedic and rehabilitation practice where Physiotherapy has a relevant role in the excellence of the final results.
